# Development of an mPBPK machine learning framework for early target pharmacology assessment of biotherapeutics

**DOI:** 10.1038/s41598-025-87316-w

**Published:** 2025-02-04

**Authors:** Krutika Patidar, Nikhil Pillai, Saroj Dhakal, Lindsay B. Avery, Panteleimon D. Mavroudis

**Affiliations:** 1 Department of Chemical and Biological Engineering, University at Buffalo, The State University of New York, Buffalo, NY USA; 2 Global DMPK Modeling & Simulation, Sanofi, 350 Water St, Cambridge, MA 02141 USA; 3 Global DMPK Innovation, Sanofi, Cambridge, MA USA

**Keywords:** Target pharmacology, Antibodies, Pharmacokinetics, High-throughput ML, Decision tree classification., Antibody therapy, Computer modelling

## Abstract

Development of antibodies often begins with the assessment and optimization of their physicochemical properties, and their efficient engagement with the target of interest. Decisions at the early optimization stage are critical for the success of the drug candidate but are constrained due to the limited knowledge of the antibody and target pharmacology. In the present work, we propose a machine learning-based target pharmacology assessment framework that utilizes minimal physiologically based pharmacokinetic (mPBPK) modeling and machine learning (ML) to infer optimal physicochemical properties of antibodies and their targets. We use a mPBPK model previously developed by our group that incorporates a multivariate quantitative relationship between antibodies’ physicochemical properties such as molecular weight (MW), size, charge, and in silico + in vitro derived descriptors with their PK properties. In this study, we perform a high-throughput exploration of virtual antibody drug candidates with varying physicochemical properties (binding affinity, charge, etc.), and virtual target candidates with varying characteristics (baseline expression, half-life, etc.) to unravel rules for antibody drug candidate selection that achieve favorable drug-target interaction, which is defined by target occupancy (TO) percentage. We identified that variations in the antibody dose and dosing scheme, target form (soluble or membrane-bound), antibody charge, and site of action had a significant effect on the TO and selection criteria for antibody drug candidates. By unraveling new design rules for antibody drug properties that are dependent on ML-based TO assessment, we deliver a first-in-class ML-based target pharmacology assessment framework toward better understanding of the biology-specific PK and ADME processes of antibody drug candidate proteins and reduce the overall time for drug development.

## Introduction

The discovery and development of first-in-class therapeutic antibodies often begins with the assessment of the physicochemical properties of the antibodies and their efficient engagement with a new target. Once a therapeutic target is selected, rational decisions must be made for lead optimization and candidate selection in the early drug development, which can decide the success and failure in clinical stages^1^. The failures in drug development process can often be attributed to insufficient specificity of a drug candidate towards its target. It is essential to perform a target pharmacology assessment of an antibody candidate at an early stage in this process. During the evaluation of a drug-target interaction, suitability for drug-target binding, feasibility, pharmacokinetics, pharmacodynamics, and safety are some of the factors that are important in understanding the drug’s pharmacology^2,3^. It is relevant to understand the magnitude and duration of target engagement to enable selection of appropriate drug candidates and identification of optimal drug-target pairs. To obtain a robust target engagement, optimization of multiple properties like antibody binding affinity, targeted human efficacious dose, and dosing regimen, is required to select the best candidate with desired biological activity^4^. The binding affinity of a therapeutic antibody candidate to its target is one of the aspects that governs the target pharmacological effect^5^. The affinity and pharmacological activity are also interrelated to the physicochemical properties of the antibodies. Antibody engineering allows controlling these properties early in the drug development process. Often, the set of desired properties related to an antibody candidate required for an optimal target engagement and pharmacological response are not clearly known in the early stages of drug discovery and development. To identify desired properties, extensive in vivo pharmacology studies for each drug candidate may be required, which can be highly resource-intensive^6^. Computational exploration of these engineered parameters before comprehensive experimental evaluation could provide useful early insights into desired properties and accelerate lead discovery.

Drug-target interactions not only depend on drug and target properties but also on physiological attributes of species^1^. It is essential to study the drug’s physicochemical (PC) and pharmacokinetic (PK) properties as well as the influence of non-specific interactions of the antibody within the targeted site of action to understand the desired target engagement response^7–10^. For instance, net surface charge and isoelectric point of antibodies lead to unintended charge-based non-specific interactions with cell components, which affects their clearance and tissue distribution, and ultimately their PK^7,9–11^. This may have an adverse effect on the desired target engagement response. Many research studies have made it evident that an integrated approach to understand the relative contribution of both PC properties and species physiology on ADME/PK properties is critical to rationally drive and inform engineering strategies to achieve the desired antibody drug-target response^1,4,12–15^. Often, physiologically-based pharmacokinetic models (PBPK) and pharmacodynamic (PD) models are considered useful in predicting target engagement response and improving confidence in early decisions^12^. Although such models are capable of early decision-making, integrating them with both in vivo pharmacology insights and high-throughput machine learning (ML) analysis can be promising and advantageous for the early drug discovery and development stages^6^. In the past, Chen et al. proposed a ML-based target pharmacology assessment to obtain an optimal combination of PK, potency, and ADME of small molecule drugs by combining a PBPK/PD/ML modeling approach^6,16^. Makowski et al. identified optimal antibody combinations of low off-target binding and low self-association using an interpretable ML approach and in vitro measurements but lacked its association with PK/TO endpoints^15^. There is still a need for an integrated modeling framework in the antibody drug research space. Such a framework can assess antibody pharmacological activity based on physiology and physiochemical properties of both drug and target to optimize antibody development as well as target engagement criteria.

In this work, we provide a framework for ML-based assessment of target engagement response of antibody drug candidates by integrating a minimal PBPK (mPBPK) model with a decision tree-based ML model. We generate a large number of virtual drug-target candidate pairs by using our previously developed mPBPK model and varying the affinity to the target, target’s baseline concentration, and turnover rate. These virtual drug and target properties are categorized into optimal or non-optimal spaces according to a desired target occupancy (TO) percentage. An interpretable decision tree-based algorithm was applied for high-throughput screening of virtual drug-target properties such as binding affinity, baseline concentration, target half-life, dose, dosing scheme, and antibody charge to identify the combination of properties that are most likely to demonstrate the desired target engagement response. To the best of our knowledge, such a ML-based target pharmacology assessment has not been performed before for antibodies. It can serve as a useful platform for high-throughput assessment of PK/TO and PD response of antibodies in the future.

## Methods

### Minimal PBPK model for monoclonal antibodies

To study the relationship between antibody properties and ADME, PBPK models are often the models of choice since they provide quantitative descriptions of the drug disposition process in a biological system that can be scaled between species based on physiological differences^12^. Previously, we developed a mPBPK model integrated with a target-mediated drug disposition model (TMDD) that quantitatively relates the PC properties of antibodies such as MW, size, charge, binding affinities to FcRn and specific targets with their ADME and PK properties^11^. The structure of the mPBPK model includes a plasma compartment, two lumped tissue compartments, mainly tight tissues and leaky tissues, and a lymph compartment^11^. Briefly, the mPBPK model takes in the physiology-based parameters (eg. organ volumes, tissue lymph flows, blood flows), antibody-specific properties (eg., MW, size, charge, binding affinity), and target properties (eg. baseline expression, half-life) as the input. The mPBPK model was fitted and validated using both in vivo mice and clinical data as available and simulated in Matlab^11^. The mPBPK model mainly incorporates the effect of FcRn and target binding, MW/size, and charge on specific and non-specific clearance and distribution of intravenously administered antibodies^11^. More detailed information on the model kinetics, parameterization, and assumptions is provided in the supplementary file.

The mPBPK model predicts the antibody PK profile, PK endpoints, and TO% for a given antibody-target pair. TO% is calculated as the ratio of the bound target over the total target, which can be calculated different compartments, mainly plasma, tight tissues, and leaky tissues (Equation S1, supplementary file). For a given antibody-target pair, we calculated TO% at the minimum and maximum concentration of antibody drug in plasma for each scenario (supplementary file, Table S1). TO% was used as a way to quantify achievable target engagement. Besides TO%, we also calculated the PK and exposure endpoints, mainly the area under the curve at steady state (${\text{AUC}}_{\text{ss}}$), minimum plasma concentration (${\text{C}}_{\text{min}}$), maximum plasma concentration (${\text{C}}_{\text{max}}$), total soluble and membrane target suppressed. However, these calculated PK endpoints and PK exposure are relevant to understanding targeted pharmacology, they were not used in building the ML model. The calculated PK endpoints (${\text{AUC}}_{\text{ss}}$, ${\text{C}}_{\text{min}}, {\text{C}}_{\text{max}}$) are not reported here. The mPBPK simulated scenarios involve three different doses, 0.1, 1, and 10 mg/kg, three different dosing regimens (once per week, once per 2 weeks, once per 4 weeks), and three different antibody charge variations (+ 5, 0, and -5). The antibody drug is administered intravenously at a fixed dose in the plasma compartment. We assume a hypothetical variation in the net surface charge on an antibody. Our mPBPK model can relate the variable region (Fv) charge on an antibody to its clearance, distribution, and non-specific cellular interaction^11^. These hypothetical charge variants are representative of charge variations in the variable domain, which is an essential property for antibody engineering^10^. We provide more details on scenarios used to simulate the mPBPK model and generate data in this study in the supplementary material. (Table S1, supplementary file).

### Virtual data generation

We used virtual data generated using a combination of properties of antibodies and targets, such as binding affinity, target baseline, and target half-life (${\text{K}}_{\text{D}}, {\text{T}}_{\text{s}0}, {\text{t}}_{1/2\text{s}}$). To understand the effect of properties of the antibody-target pairs on targeted pharmacology, we used the mPBPK model to calculate the TO% (Equation S1, supplementary file).

We used a log-normalized uniform sampling method (scipy.stats.loguniform) to generate 10,000 virtual candidates to perform the ML-based assessment. 10,000 candidates provide a balance between computational costs and capability of the machine learning model for generalizing well for different scenarios. To account for a wide range of possible target characteristics, the virtual target candidates were generated with varying target baseline values (1 pM – 1000 nM) and target half-life (1 min – 300 h) for both soluble and membrane-bound receptors. Similarly, the virtual drug candidates were generated with varying drug-target binding affinity (1 pM – 1000 nM). Each virtual combination of drug and target candidate denotes a unique drug-target interaction. In our case, 10,000 virtual candidates were considered appropriate based on a comparison of decision tree-based decision rules and decision tree model performance (Table S4, supplementary file). The generated data was categorized into two classes based on TO% > 90% or < 90% at ${\text{C}}_{\text{min}}$ in plasma at steady state. The class distribution in the virtual data was highly imbalanced. Therefore, we applied oversampling of the minority class in our imbalanced classification dataset using Synthetic Minority Oversampling Technique (SMOTE) as part of the imbalanced-learn package in Python^17^. SMOTE works by selecting examples that are close to the feature space, drawing a line between the examples in the feature space, and drawing a new sample at a point along that line^17^. This procedure was used to create as many synthetic examples for the minority class as observed in the majority class. After balancing the class for each dataset, we obtained a dataset with a total size of slightly over 10,000 data points for each scenario. For instance, if a dataset contained 5452 data points in the majority class, SMOTE oversamples in the minority class to get 5452 data points in the minority class, which results in a total size of 10,904 data points. The SMOTE-balanced data was assumed as the ground truth in this work. The virtual antibody and antigen candidates within the balanced dataset were considered unique.

### Decision tree-based supervised machine learning

A supervised classification model was applied to categorize the virtual candidate data. An interpretable decision tree-based algorithm was trained and evaluated using the virtual data for each scenario (Table S1, supplementary file). Each drug-target candidate pair was classified based on TO% calculated at ${\text{C}}_{\text{min}}$ into an optimal class ($>$ 90%) or non-optimal class ($\le$ 90%). We used 90% of the SMOTE-balanced dataset for training the classifier and reserved 10% for testing. A subset of training data used to train the ML model is shown as an example in the supplementary file, Table S5. We used decision-tree-based classifier as the algorithm for the following reasons. We compared different tree-based algorithms in the scikit-learn package in Python, namely decision trees, random forest, and gradient boosting algorithms. We compared the mean training accuracy, precision, and F1 score for five-fold cross-validation across the three models for a fixed dose of 0.1 mg/kg (supplementary file, Table S6). We also compared these metrics on the test dataset across each model (supplementary file, Table S6). Decision trees, random forests, and gradient boosting classifiers had comparable performance. However, decision tree classifiers were computationally less expensive relative to other classifiers, which was based on the computing time. Therefore, decision tree-based classifiers were used for different scenarios throughout this study. However, model performance could be different for other scenarios and datasets.

We used a Decision tree classifier (*decisiontreeclassifier)* from *sklearn* package in Python 3 using default hyperparameters, except *max_depth* = *5, criterion* = *‘gini’, min_samples_split* = *3, splitter* = *‘best’, class_weight* = *‘balanced’,* which were obtained from a hyperparameter search using gridsearchCV*.* We used 90% of the balanced dataset for training and the rest as the test dataset. We trained the decision tree classifier using a stratified five-fold cross-validation for bi-label classification based on TO%. Additionally, we also performed a three-label classification using decision trees on the virtual dataset, which was generated for a fixed dose of 1 mg/kg Q2W (IV). We classified the decision tree-based rules for drug-target properties for a low ($\le$ 50%), medium (50%—90%), and high TO% ($>$ 90%). The decision tree-based predictions were visualized using the Matplotlib and Seaborn packages in Python^18,19^.

### mPBPK / ML model framework

**Fig. 1:**
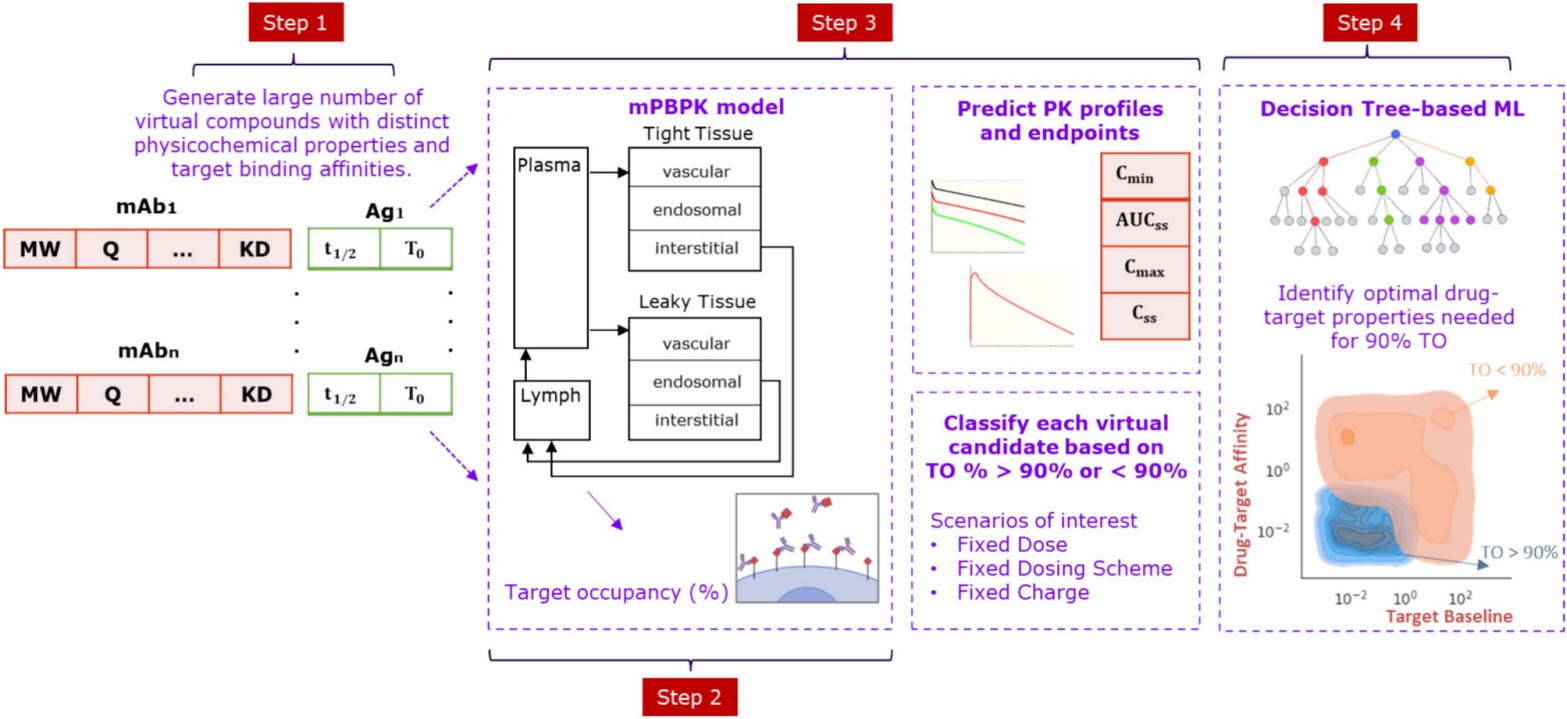
Representation of steps involved in the development of the minimal physiologically based pharmacokinetic / Machine learning modeling framework. mAb: monoclonal antibody, Ag: Antigen or target, MW: Molecular weight, Q: antibody charge, KD: Binding constant, t_1/2_: target half-life, T_0_: target baseline, TO: target occupancy, C_min_: minimum plasma concentration, AUC_ss_: area under the curve at steady state, C_max_: maximum plasma concentration, and C_ss_: plasma concentration at steady state.

We built a ML-based target pharmacology assessment framework that combines a mPBPK model^11^, a TMDD model^11^, and an ML model to infer the optimal drug and target characteristics responsible for desired target engagement and occupancy. Our ML-based framework was implemented to study the relationship between PC properties of the antibodies like charge and drug-target binding (${\text{K}}_{\text{D}}$), ADME characteristics, and target properties like baseline (${\text{T}}_{0}$), half-life (${\text{t}}_{1/2}$), and soluble or membrane-bound forms of the target. Figure. 1 shows the steps involved in the model development process. Firstly, we generated many virtual candidate pairs of antibody drugs and their specific targets. Each virtual candidate signifies a unique drug-target interaction. A virtual drug candidate was generated by varying dose level, dose regimen, surface charge, and target binding affinity. A virtual target candidate was generated by varying target baseline expression and target turnover or half-life. Secondly, we used the mPBPK model to simulate the PK profile and PK/exposure endpoints (${\text{AUC}}_{\text{ss}}, {\text{C}}_{\text{min}}, {\text{C}}_{\text{max}}\text{ etc}.$) for each virtual candidate^11^. We assess the magnitude of target engagement response for each virtual candidate by calculating the TO percentage (TO%). A decision tree based ML algorithm is used to classify each virtual candidate (or drug-target interaction) into an optimal or non-optimal interaction based on the calculated TO%. The ML classifier helps to infer the optimal combination of drug and target properties that is more likely to provide the desired TO endpoint and eventually desired pharmacological effect.

## Results

**Fig. 2:**
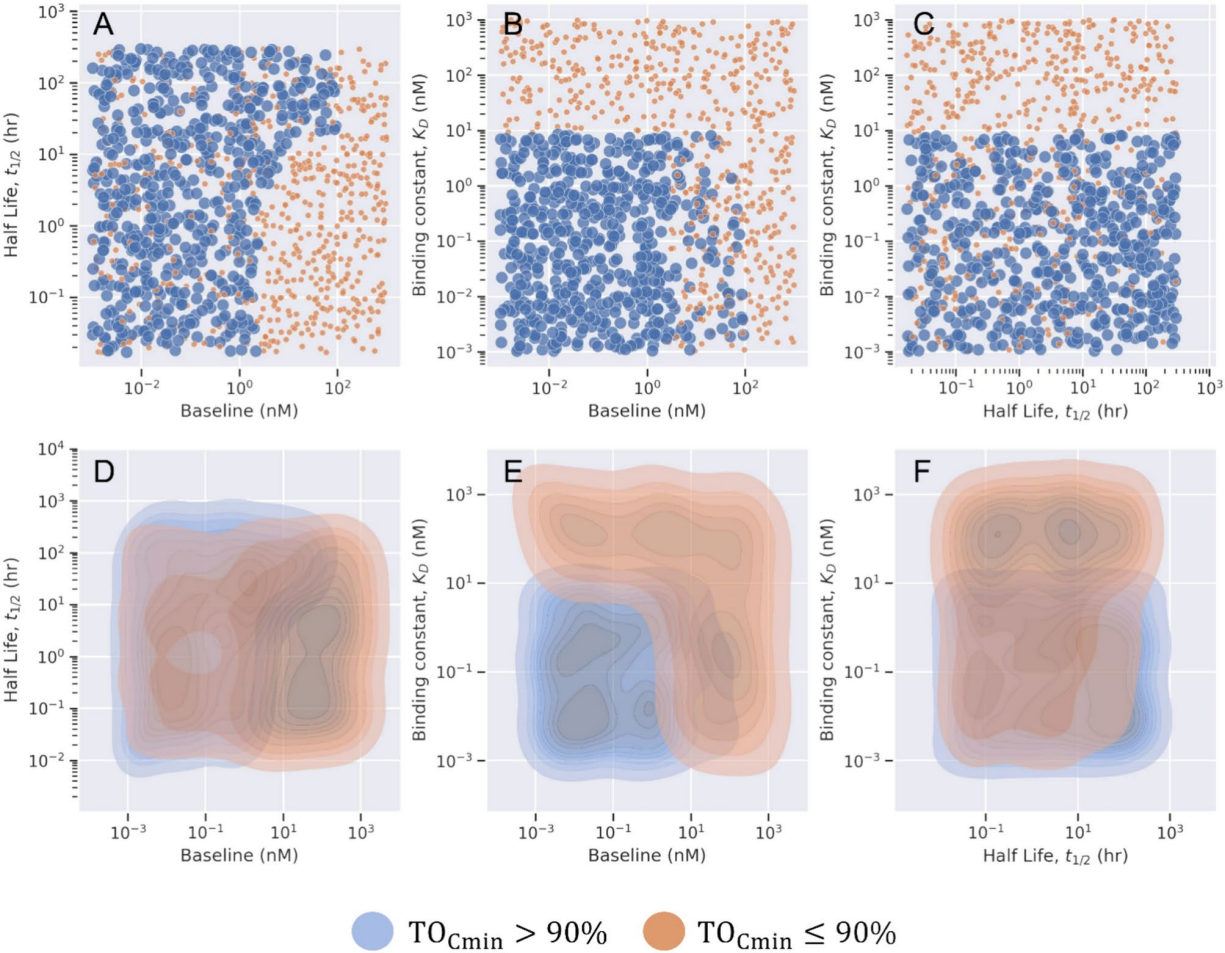
Representation of results from a specific scenario. Antibody is administered at 1 mg/kg through intravenous (IV) route once every two weeks. Target Occupancy percentage (TO %) is measured in plasma for drug and soluble target engagement. The pairwise scatter plot (top panel) and pairwise Kernel density plot (bottom panel) shows the pairwise distribution of properties (baseline, binding constant, and half-life). Each property is classified into optimal (blue) and non-optimal class (orange) based on TO% calculated at minimum concentration at steady state.

We used the virtual data to train the ML classifier for different dose variations, dosing frequencies, antibody charge variations, and across different forms of target (soluble or membrane-bound). A complete list of scenarios considered to generate data for ML model development is provided in Table S1 the supplementary file. In Fig. 2, we provide the results from the decision tree-based model using 10,000 virtual drug-target candidates, where the antibody was administered via intravenous (IV) route at 1 mg/kg once every 2 weeks (Q2W) and bound to only soluble forms of target. The decision tree model uses the appropriate cut-off value for each input feature (${\text{K}}_{\text{D}}, {\text{T}}_{\text{s}0}, {\text{t}}_{1/2\text{s}}$) to classify each virtual antibody drug-target candidate into an optimal (blue) or non-optimal (orange) class based on the TO% observed at the minimum concentration in plasma at steady state. The decision tree-based model suggests that drug administered at 1 mg/kg once every two weeks should have greater than 90% target engagement when the binding constant (${\text{K}}_{\text{D}}$) is below 9 nM for a class of soluble targets that have baseline values below 4 nM in plasma (Fig. 2). The decision tree-based rules for optimal class prediction were chosen based on Gini impurity close to 0 and accuracy close to 100%. It is important to choose the best rule with the highest accuracy as it increases the likelihood of true prediction for other unseen datasets. ${\text{K}}_{\text{D}}$ and ${\text{T}}_{\text{s}0}$ are the most important properties to predict more than 90% TO as a representative target engagement criterion. ${\text{t}}_{1/2\text{s}}$ parameter has the least influence in decision making in this scenario. However, the class of soluble receptors with low half-life and high baseline may be the least ideal combination of target properties to achieve greater than 90% TO in plasma (Fig. 2). The binary classification decision tree for the above scenario is provided in Figure S1 in the supplementary file. Each decision tree-based model was evaluated on a separate validation data set based on the following evaluation metrics such as classification metric, confusion matrix, and area under the receiver-operating curve (ROC) (Figure S1, supplementary file). The set of optimal and non-optimal rules used in decision tree-based binary classification for this scenario are also provided in the supplementary file.

### Optimal drug-target properties for target engagement for different doses and regimens

**Fig. 3:**
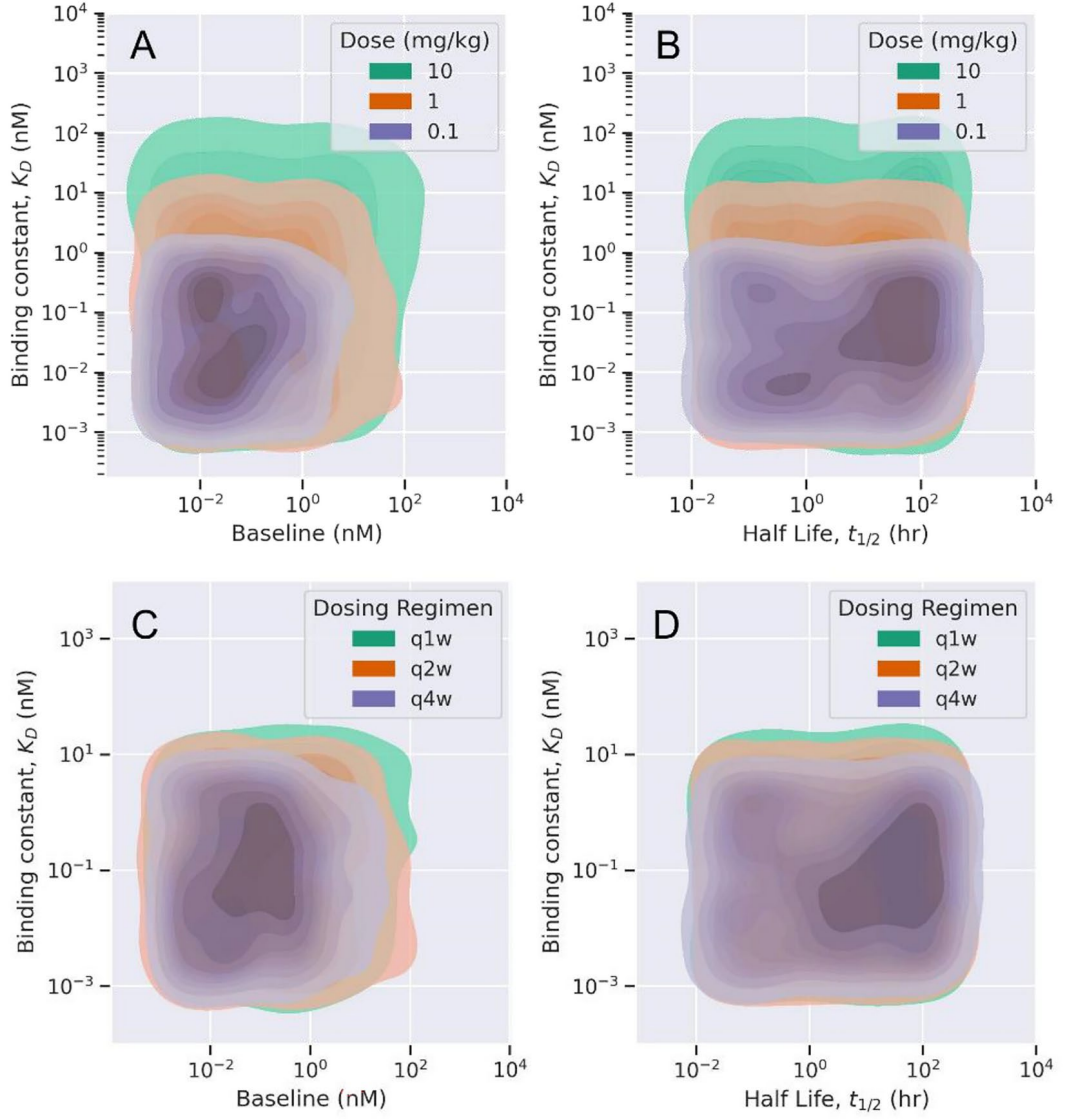
The effect of different doses (A, B) and dosing schemes (C, D) on drug-target properties for optimal target engagement. (A, B): The tree-based ML model was trained and validated for synthetic data based on different doses of 0.1, 1, and 10 mg/kg administered once every 2 week (Q2W). (C, D): The tree-based ML model was trained / validated for synthetic data based on a fixed dose of 1 mg/kg administered once every week (Q1W), once every two weeks (Q2W), and once every 4 weeks (Q4W). Only optimal properties dependent on target occupancy > 90% at minimum plasma concentration at steady state are shown.

Fig. 3 shows the difference in optimal rules and values of drug-target properties for different doses and dosing schemes. The decision tree-based ML model provided an optimal region for drug-target binding constant (${\text{K}}_{\text{D}}$), target baseline, and target half-life values for bolus and repeated IV doses of 0.1, 1, and 10 mg/kg (Fig. 3). The optimal rule for a low dose (0.1 mg/kg) scenario suggests a ${\text{K}}_{\text{D}}$ value below 1 nM. A drug administered at such a low dose achieves 90% TO with class of soluble receptors with baseline values below 2 nM (Fig. 3). As the dose is increased from 0.1 mg/kg to 1 mg/kg, the cut-off for optimal ${\text{K}}_{\text{D}}$ value increases to 9 nM as well as target baseline values increase to 4 nM (Fig. 3). Similarly, at high dose of 10 mg/kg, the optimal cut-off for ${\text{K}}_{\text{D}}$ increased to 82 nM. At a lower administered dose the drug clears faster and undergoes target-mediated disposition. Therefore, to achieve greater than 90% TO at such a low dose, drug-target affinity should be higher (${\text{K}}_{\text{D}}$ values should be lower). The optimal rules suggested that ${\text{K}}_{\text{D}}$ and target baseline were relatively more important to classify a given drug-target pair based on target engagement response. It is known that target baseline concentration and target half-life are relevant target properties that govern drug-target interaction and desired target pharmacology^1,13^. However, to achieve more than 90% TO at different doses, the target half-life was the least influential in the decision-making process. On the other hand, we also observed the effect of the dosing scheme on optimal TO% when the drug is administered at a repeated dose of 1 mg/kg once every 1 week (Q1W), once every 2 weeks (Q2W), and once every 4 weeks (Q4W) (Fig. 3). The decision tree-based rules deemed that as dosing frequency increases, the optimal range for ${\text{K}}_{\text{D}}$, ${\text{T}}_{\text{s}0},$ and ${\text{t}}_{1/2\text{s}}$ increases. The effect of dosing frequency on optimal drug and target properties can be observed in the density plot, Fig. 3. For Q4W, Q2W, and Q1W dosing scheme, ${\text{K}}_{\text{D}}$ values below 4.25, 9, and 15. 4 nM will provide greater than 90% TO for a given class of soluble receptors with baseline values below 2, 4, and 8.3 nM, respectively. Target half-life has the least dependence on the dosing scheme and was insufficient in solely determining the optimal class.

### Effect of antibody charge on optimal drug-target engagement

**Fig. 4:**
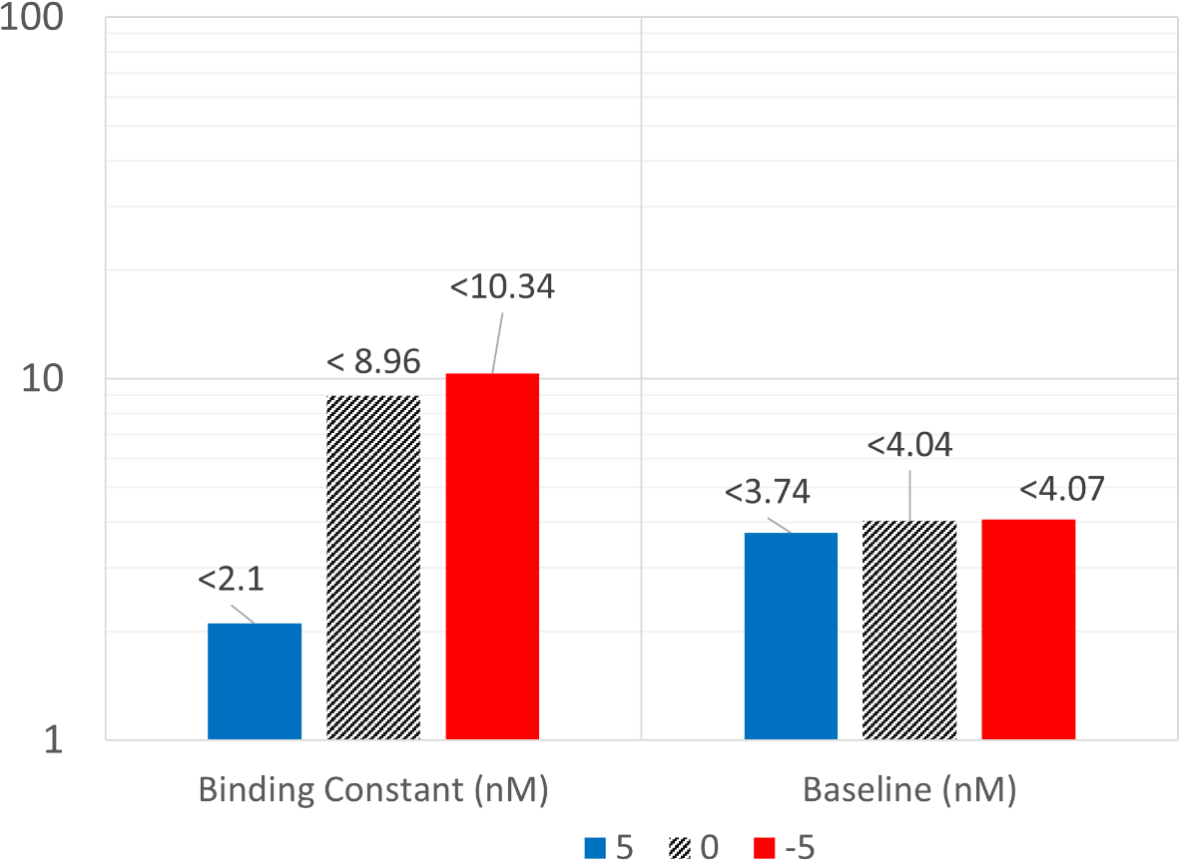
The effect of antibody charge on optimal drug and target properties needed for optimal target engagement response. The bar plot represents the maximum cut-off values of binding constant (nM) and target baseline (nM) as predicted by the tree-based classifier needed to achieve TO% > 90% in plasma. Each antibody has a net surface charge of + 5, 0, and -5, and is administered at 1 mg/kg once every 2 weeks.

The net surface charge, charge distribution, and isoelectric point (pI) of an antibody affect nonspecific cellular uptake and degradation^10^. The surface charge of a therapeutic protein is a property of the amino acid sequence of the protein and the pH of its surroundings^20^. The net surface charge leads to non-specific interactions with the charged extracellular matrix components and membrane proteins in the cells, resulting in enhanced pinocytotic uptake and degradation, suggesting that charge can be a relevant descriptor of antibody ADME processes^21^. Hence, charge or isoelectric point is a relevant and inherent property of an antibody which can affect its specificity and target engagement response. We incorporated the effect of charge in the decision tree classifier and observed a significant contribution on optimal (> 90%) TO (Fig. 4). Figure 4 quantifies the best ML-derived rules. ${\text{K}}_{\text{D}}$ values should be lower than 2.1 nM for when the targeted class of soluble receptors have a baseline below 4 nM approximately for relatively positively charged antibody. Similarly, the cut-off for ${\text{K}}_{\text{D}}$ values increased with a more negative charge on the antibody. This agrees with our previous findings from the mPBPK model, where a positive charge on an antibody had a significant effect on clearance and distribution^11^. A positively charged antibody with faster clearance has relatively lower exposure in plasma and restricts the amount and duration of engagement with its specific target. Also, at lower baseline values of a given soluble target, half-life did not affect rule-based classification (Figure S2, supplementary file). However, if the potential soluble targets have a higher baseline expression, it is critical to account for the target half-life (Figure S2). A high baseline and longer half-life of a target is an optimal combination of target properties to achieve high TO%.

### Assessment of target form on optimal target engagement in Tissues

**Fig. 5:**
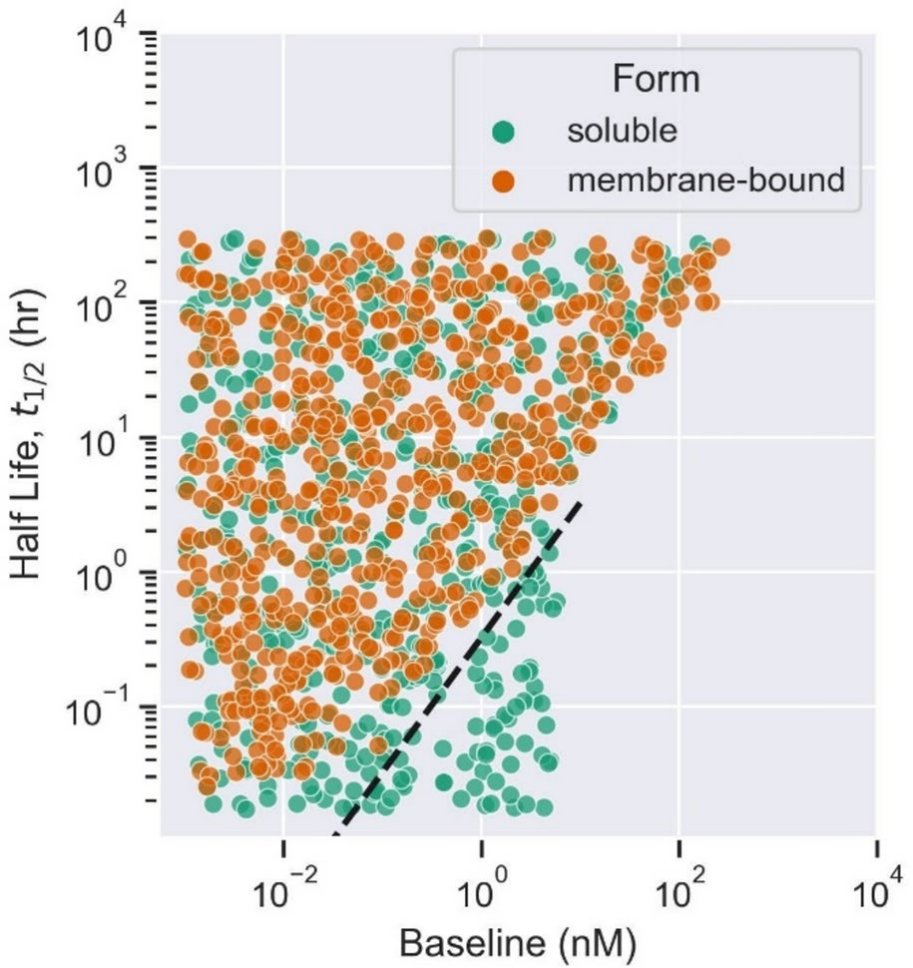
The effect of different forms of target, soluble (green) or membrane-bound (orange). The decision tree-based boundaries for target properties (half-life and baseline) needed for optimal target occupancy (TO% > 90%) for each target form are shown. The fitted dashed black line (y = 0.324x) separates the optimal properties needed for soluble and membrane-bound receptors that achieve 90% target occupancy.

We simulated the mPBPK model for two different forms of target (soluble or membrane-bound). Virtual drug-target candidate properties were the same for the above simulations. The predicted TO% at the minimum concentration in leaky tissues was used as a criterion for binary classification. The optimal TO% (> 90%) for antibody engagement with soluble target and membrane-bound target are shown in Fig. 5. The scatter plot (Fig. 5) shows the optimal values of target baseline and half-life to achieve TO% > 90% in plasma with soluble and membrane receptors. Rules indicated that for a given drug candidate with optimal ${\text{K}}_{\text{D}}$, a class of membrane-bound receptors with a baseline value below 4 nM and a half-life greater than 20 min is suitable. However, for a given drug candidate with optimal ${\text{K}}_{\text{D}}$, a class of soluble receptors with a baseline value below 7 nM is more suitable, whereas half-life doesn’t affect the target candidate selection. We also observed a separation in property values (baseline, half-life) between soluble and membrane-bound receptors that achieve more than 90% TO. A dashed line was fitted with target properties at this separation boundary (Fig. 5). The combination of target baseline and half-life values below the dashed line are optimal for soluble targets. The target baseline and half-life values within the area bounded by the fitted line and target baseline cutoff value (< 7 nM) for soluble receptors are most likely to achieve TO% > 90%. The membrane-bound complex internalizes at the same rate as the target degradation, which is dependent on the target half-life. Therefore, a lower half-life means a higher target degradation and higher internalization rate of the drug-target complex. Both target degradation and complex internalization affect the desired TO%, as shown in Equation S1 in the supplementary file. The soluble drug-target complex internalizes at the same rate as drug clearance, which is a likely reason for observed differences in the distribution of optimal properties between soluble and membrane-bound receptors. Moreover, in each scenario, when the target baseline is high and the half-life is low, neither soluble nor membrane-bound drug-target engagement is likely to achieve greater than 90% TO (Fig. 5). The decision tree-based rules suggested slight differences in drug-target binding constant (${\text{K}}_{\text{D}}$) values between soluble and membrane-bound receptors. ${\text{K}}_{\text{D}}$ must be lower than 11 nM and 9 nM to achieve more than 90% TO with soluble and membrane receptors, respectively.

### Optimal drug-target properties for target engagement at different sites of action

**Fig. 6:**
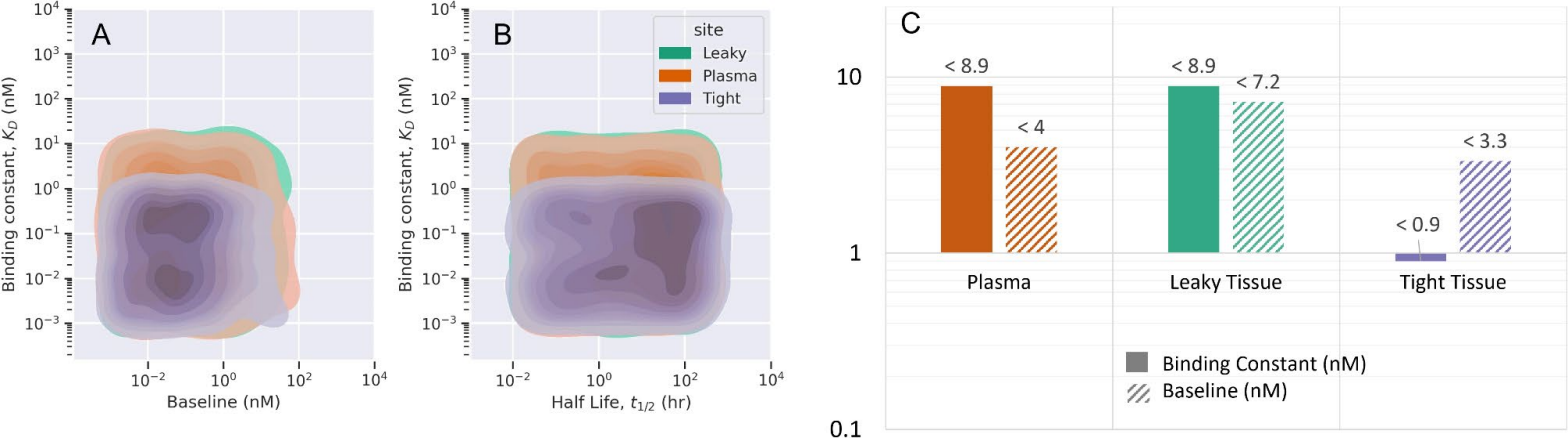
The ML-derived rules for optimal properties for different sites of action (plasma, leaky tissues, tight tissues). The distribution of drug properties (binding constant, ${\text{K}}_{\text{D}}$ (nM)) and target properties (half-life, ${\text{t}}_{1/2}(\text{hr})$, baseline, ${\text{T}}_{0}$ (nM)) is provided as a pairwise kernel density plot (A, B). The ML-derived rules are quantified as optimal cut-off values for binding constant and target baseline for each site of action (C).

The drug-target interaction and engagement are influenced by the physiology and local microenvironment. The administered drug may be required to bind to receptors in different sites in the tissues besides plasma. Depending on the disease and mechanism of action, it may be useful to identify the PC properties of the antibody and specific receptors that affect drug-target interaction at different sites of action. In this study, we considered three sites of action, plasma, leaky tissue vasculature, and tight tissue vasculature. A separate decision tree-based ML model was trained for virtual data generated for the above scenarios. The TO% at the minimum concentration in plasma, leaky tissues, and tight tissues is used as a criterion for optimality for respective sites of action. The ML-derived rules suggest that stronger binding affinity (lower ${\text{K}}_{\text{D}}$ values) is suitable to achieve optimal TO% in the tight tissues compared to leaky tissues and plasma. The drug distribution and exposure in the tight tissues are relatively lower than leaky tissues and plasma. In tight tissues, the drug binds to a soluble target with lower baseline values (< 3 nM) and binding constant below 1 nM. In leaky tissues and plasma, the drug binds to a soluble target with a binding constant below 9 nM. We found no significant difference in ${\text{K}}_{\text{D}}$ values for optimal TO% in plasma and leaky tissues. We observed a relatively lower clearance and higher accumulation of the drug in leaky tissues than in plasma. This observation is also evident through the bar plot (Fig. 6). The drug has sufficient engagement with a target with baseline below 7 nM in leaky tissues and below 4 nM in plasma, which is dependent on the faster drug clearance in plasma.

**Fig. 7:**
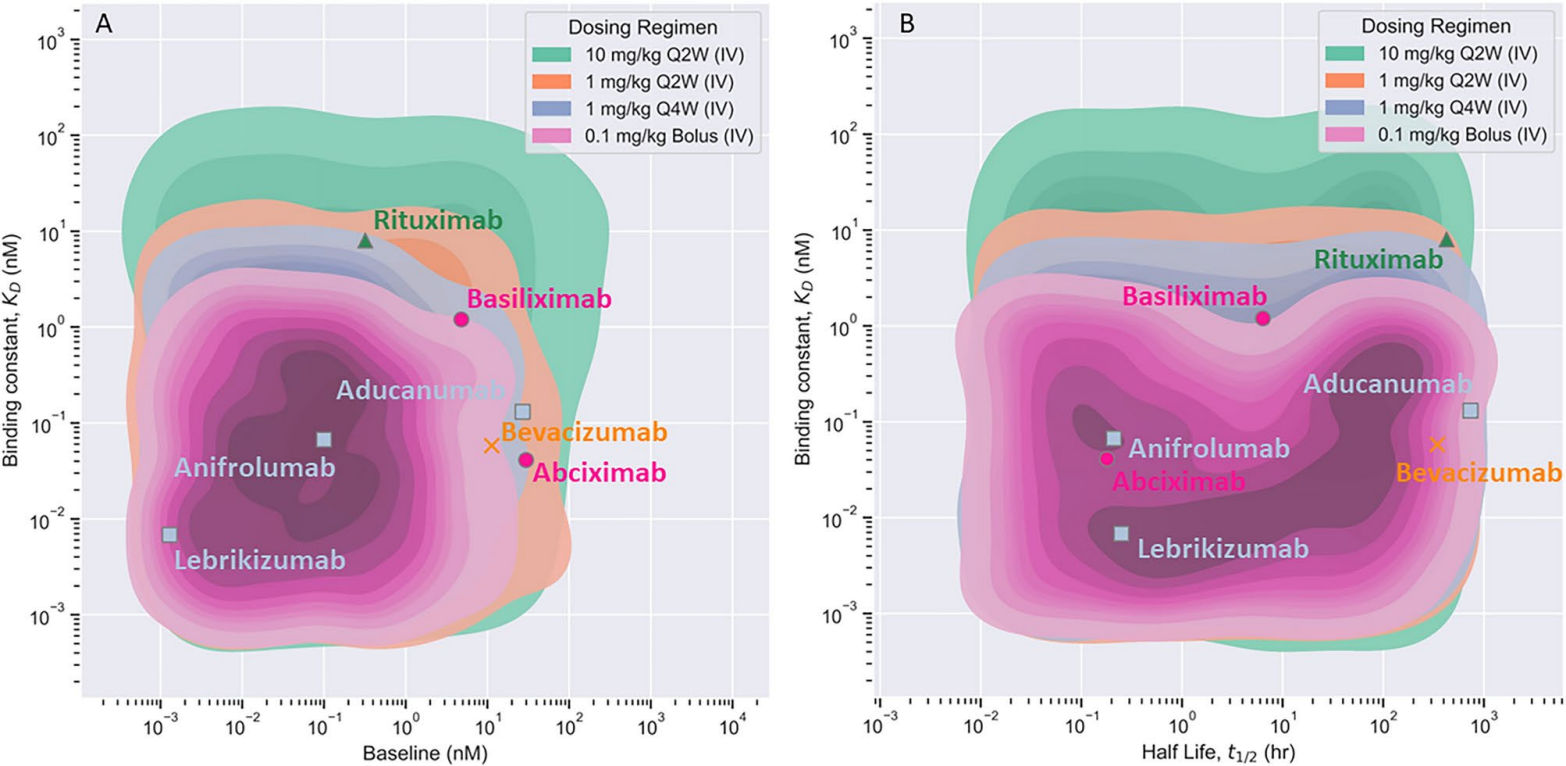
The ML-derived optimal (TO% > 90%) properties for dose of 0.1 mg/kg bolus (pink region), 1 mg/kg Q4W (IV) (blue region), 1 mg/kg Q2W (orange region), and 10 mg/kg Q2W (green region) are shown. Experimentally derived properties of clinically approved monoclonal antibodies for the respective dose regimen are shown as data points ^24–31^. The antibody data is color-coded based on dosing regimen.

The ML-derived rules for different scenarios were tested using the mPBPK model and Monolix/Simulx model ^22,23^ (Table S2). A combination of optimal and non-optimal drug and target properties were chosen based on decision tree-based rules for different scenarios such as dose, dosing scheme, etc. The mPBPK model predicted TO% accurately for all combinations used for validation as shown in Table S2 in the supplementary file. The Monolix/Simulx model^22,23^ also predicted correctly for most combinations (Table S2). The validation of ML-derived rules against the mPBPK model is expected as virtual data was generated using the mPBPK model. However, the validation of these rules against Monolix/Simulx^22,23^ predictions is also relevant as the model structure and assumptions of the Monolix/Simulx model^22,23^ may be different from the mPBPK model. These validations increase confidence in the robustness and performance of the ML model. We also validated the ML-derived rules using real data for clinically approved monoclonal antibodies. Table S3 in the supplementary file provides a list of literature-derived properties of monoclonal antibodies and their respective target, as well as recommended clinical dose and regimen^24–33^. In our study, the trained ML model can make predictions for a fixed dose and regimen administered intravenously. Considering this limitation of the ML model, we made some necessary but reasonable assumptions to validate our model against real data. We assumed a dose of for a dose of 0.1, 1, or 10 mg/kg that was nearest to the recommended clinical dose for each antibody. These assumed dose values for each antibody are provided in Table S3 in the supplementary file. We used the trained ML model to predict the optimal ${\text{K}}_{\text{D}}, {\text{T}}_{0}, {\text{and t}}_{1/2}$ values for each antibody at the assumed dose and recommended regimen administered intravenously. The optimal drug-target properties were validated for the following scenarios, 0.1 mg/kg bolus, 1 mg/kg Q4W, 1 mg/kg Q2W, and 10 mg/kg Q2W (Figure 7). The experimental properties of most antibodies lie within the predicted optimal boundaries of drug-target properties when administered at their respective dose and regimen. Except for Abciximab and its target, ${\text{K}}_{\text{D}}\text{ and }{\text{T}}_{0}$ values lie outside the predicted optimal bounds (Fig. 7A). We believe this is because the assumed dose is lower than the clinical dose (0.25 mg/kg bolus). It is likely that the optimal values of ${\text{K}}_{\text{D}}\text{ and }{\text{T}}_{0}$ will be higher for a dose greater than 0.1 mg/kg, as seen from the dose-dependence in Fig. 3 Moreover, the optimal ${\text{t}}_{1/2}$ values for each scenario (Fig. 7A) were validated very well against the experimental observation for each antibody. While these preliminary validations are helpful in validating the ML-derived rules, more data for antibody and target can help increase confidence in ML predictions.

**Fig. 8:**
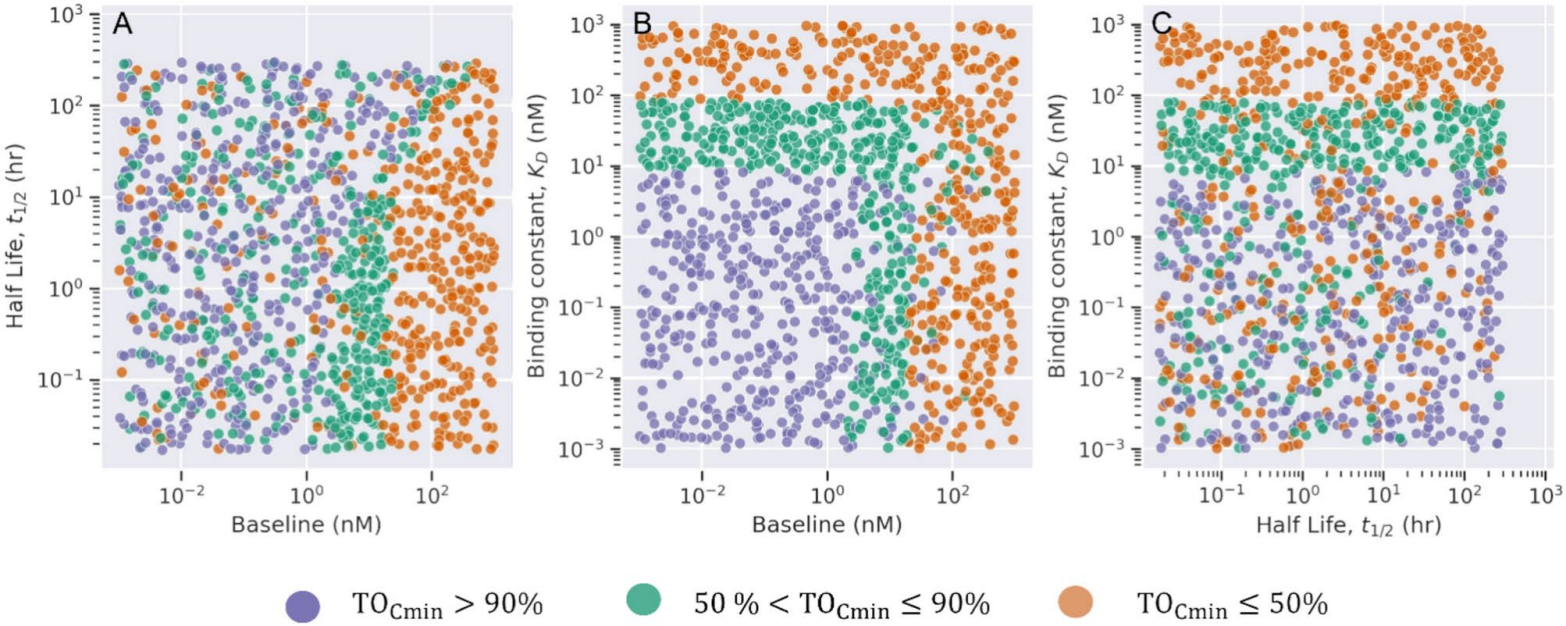
Multi-label classification of drug-target properties. Pairwise scatterplot of drug and target properties classified based on target occupancy % (TO%) <  = 50% (orange), 50–90% (green), and > 90% (purple).

The optimality criterion for TO may vary across diseases and treatments. The threshold for TO% can be different than 90%. The ML model can be modified to accommodate such differences. As an example, we performed a multi-label classification of drug-target properties based on a low (< 50%), medium (50–90%), and high (> 90%) TO. We used virtual data for drug administration at 1 mg/kg once every two weeks (Q2W) and categorized the data into three classes based on TO% at minimum concentration in plasma at steady state. The performance metrics for multi-label classifier are provided in the supplementary file (Figure S3). Figure 8 shows the optimal region for selection of drug-target binding constant for a given target with baseline and half-life that lead to low, medium, and high TO. We observed clear boundaries for separation of virtual drug-target properties for respective classes. Our results agree that higher binding affinity (lower ${\text{K}}_{\text{D}}$ values) is suitable to achieve higher TO%. Optimal ${\text{K}}_{\text{D}}$ values should be below 9 nM to achieve > 90% TO and must be between 9 and 82 nM to achieve 50–90% TO. A high drug-target engagement can be achieved with respective optimal ${\text{K}}_{\text{D}}$ values when soluble target baseline is below 24 nM. A moderate target engagement response can be expected when soluble target baseline level is below 24 nM and half-life is below 19 h. An insufficient and low target engagement response can be expected when target baseline expression is more than 24 nM and half-life is between 32–300 h. The multi-label decision tree classifier had an accuracy of 99%, and class precision of 99%, 98%, and 99% for low, medium, and high classes, respectively (Figure S3, supplementary file).

## Discussion

Antibodies represent a diverse array of large molecules that have revolutionized the treatment of a wide range of diseases and have several advantages over small-molecule drugs such as high specificity, high tolerability, and longer half-lives^34,35^. Despite recent progress, only a small percentage of drug candidates in development enter the clinic and ultimately reach the market, often due to lack of desired pharmacological activity^36^. To assess the desired pharmacology of an antibody candidate at an early stage in this process, drug-target interaction, suitability for drug-target binding, feasibility, PK/PD, and safety should be taken into consideration^2,3^. The PC properties of antibodies such as size, charge, and drug-target binding affinity influence their ADME, PK, and ultimately their desired pharmacological response^21^. Antibody engineering allows optimization of these properties early in the drug development process. In the past few decades, computational modeling techniques such as PBPK and PKPD modeling have supported drug design and development significantly^6^. In addition, ML has found its place in supporting the drug development pipeline in numerous ways^6,15,16,37,38^. In this study, we present a ML-based target pharmacology assessment framework that uses both minimal PBPK and ML modeling to determine PC properties of antibodies and specific target characteristics relevant to achieving a desired target engagement response. It is critical to understand the magnitude and duration of target engagement to enable the selection of appropriate drug candidates. We successfully achieved such a relationship by first simulating the mPBPK model with many virtual drug and target candidates with varying properties and then using a decision tree-based ML classifier to unravel rules for these properties based on a TO% as a criterion. We used a decision tree-based classification model due to its interpretable nature; it draws a relationship between input (drug-target properties) and output (TO%) using recursive partitioning in a tree-like manner (Figure S1). This interpretable ML model delineates the process of obtaining the predicted class from the given input, which makes it preferable over other black-box ML algorithms such as artificial neural networks^6,39^.

The results from our proposed ML-based framework showed a quantitative relationship between antibodies’ PC properties on ADME, PK, and TO for different intravenous doses (Fig. 3a,Fig. 3b), dosing schemes (Fig. 3c,Fig. 3d), and at different sites of action (Fig. 6). We found that decreasing the dose and/or frequency of dosing necessitates the selection of antibody candidates with stronger binding affinity with a class of specific receptors. Increasing dose and frequency of administration results in higher concentration and accumulation of the drug in both plasma and tissues. To achieve greater than 90% TO, the model also presented rules for antibody candidate properties and target candidates that were different depending on the site of action, such as plasma, leaky tissues, or tight tissues. Our results revealed that optimal rules are further dependent on molecule’s charge (Fig. 4), target’s characteristics such its baseline and half-life Fig. 5 and whether the target is expressed in leaky or tight tissues (Fig. 6). Furthermore, our framework indicates that targets’ baseline and half-life requirements for efficient binding for a certain dose and regimen, is different between soluble and membrane-bound targets a dynamic that points towards different elimination mechanisms between the different targets’ forms (e.g. target-mediated clearance vs plasma clearance). To sum up, our high-throughput screening technique allows us to narrow down the drug candidates with the highest potential based on a combination of their structure-based properties, ADME, PK, and TO. For instance, the ML model reduced the initially available search space for antibodies from a total of 11,620 candidates to 590 optimal candidates based on TO%, which is approximately 5% of the total virtual candidates. The decision tree-based ML is robust and reasonably accurate when validated against traditional model-based results and observed data.

### Applications to early stages of antibody discovery and development

The proposed mPBPK/ML modeling framework would enable researchers to screen and optimize thousands of antibody candidates based on optimal ADME, PK, and drug engagement to specific targets, and reduce attrition rate and overall discovery time for lead candidates. To speed up hit-to-lead, this work can identify which candidate hits to move forward. Such a high-throughput screening and evaluation of antibody properties has not been done previously and will allow candidate evaluation against TO and engagement very early in the drug discovery and development pipeline. The outcome from the mPBPK/ML modeling framework would provide scientists with optimal starting values and a range of properties for antibody engineering/development.

The modeling framework can be used for the following potential applications and future improvements. For target selection, the ML-based prediction of the optimal bounds could assist scientists in selecting the desired target with optimal properties. For lead candidate generation, this framework can be used to determine optimal candidate properties for desired target engagement that are needed to progress from the hit to lead stage. These advanced high-throughput screening techniques enable scientists to narrow down the candidates with the highest potential based on a combination of their structure-based properties, ADME, PK, and TO. The mPBPK/ML framework provides a tabulated list of combinations of drug candidate properties and specific target properties to scientists for all the candidates that achieve greater than 90% target engagement, which can be used by scientists as an initial reference sheet to focus on the candidates with the most potential. Chemists can use this framework for a priori knowledge of the dose-driven design of antibodies, to prioritize the candidates with the highest potential to achieve target engagement and enter lead stage at the lowest dose. The mPBPK/ML framework can also be used by teams for in vitro ADME screening efforts during hit-to-lead or early lead optimization, and visualization of the optimal spaces of candidate properties to define the most critical combination of properties for screening^6,34^. The mPBPK/ML framework can identify optimal drug-target pairs based on drug-target engagement and interaction, identification of these drug-target pairs has many benefits such as drug repositioning^40,41^. ML models for the prediction of drug-target interaction pairs have been done previously based on chemical structure or sequence and not necessarily based on molecular properties and TO%^40,41^. Further, we aim to explore more advanced interpretable ML algorithms. The current framework serves as a foundation that can incorporate, as additions, other in vitro or in silico derived properties of the antibodies, other PK endpoints such as clearance, pharmacodynamic effect, and safety to train the ML model.

In conclusion, the proposed framework does not aim to replace classical model-based PK/PD or PBPK/PD assessments, but rather to be used as an early drug-target property investigation tool in research when no mechanistic insights of the drug are available. As antibodies proceed to later stages of drug discovery and more knowledge becomes available for their mechanism of action or disease of interest, a dedicated PK/PD – PBPK/PD model needs to be implemented to enhance decision making.

### Challenges and limitations

The proposed modeling framework has several challenges and some limitations that can be overcome with necessary data availability and improvements. Currently, the validity and robustness of the ML outcome depend on the accuracy of the mPBPK model, which provides the necessary PK/TO output for respective virtual candidate properties. At the early stages of drug discovery, a detailed experimental validation of target interaction, TO%, and the drug’s mechanism of action may not be available and must rely on model-based prediction. Moreover, our framework is also limited to an assessment of drug properties against a PK or TO endpoint and does not incorporate the PD effect and efficacy of the drug. The desired TO is very much dependent on the target of interest and can be variable among different diseases and disease states. In this work, we used a typical value of 90% TO, which is occasionally used as a typical value indicating efficacy when no other information is available. Despite this limitation, our results were able to capture optimal ranges from various compounds targeting different targets for various diseases (Fig. 7). Despite this limitation, the mPBPK/ML model identifies candidate properties that achieve the desired target engagement response and indicates bounds for different properties to achieve the same desired effect at a reduced dose, dosing frequency, etc. The preliminary analysis for early discovery of antibodies using virtual data and ML-based TO endpoints should be reasonable to evaluate potential candidates. The mPBPK/ML model may further be improved to use other PK endpoints such as clearance, potency, etc. as criteria to identify optimal properties based on the research question at hand. The quality and accuracy of the mPBPK/ML model should also improve with more scientific investment in early discovery efforts. With advancement in the early discovery pathway, the modeling framework can be updated and re-trained as new data becomes available.

In conclusion, we deliver a first-in-class ML-based target pharmacology assessment framework that integrates mPBPK and ML model to better understand the biology-specific PK and ADME processes and desired target pharmacology of monoclonal antibodies. By unraveling new ML-based design rules for the selection of drug and target properties based on target engagement response, we can reduce the overall time for drug candidate selection in the early discovery stages.

## Supplementary Information

Supplementary Information.

## Data Availability

The virtual datasets generated during and/or analyzed during the current study are available in public GitHub repository (https://github.com/Sanofi-Public/DMPK-US-ML-mTPA).

## Code availability

The computer codes used for this study have been deposited in GitHub (https://github.com/Sanofi-Public/DMPK-US-ML-mTPA).
