# Supplementary material for: Development of an mPBPK machine learning framework for early target pharmacology assessment of biotherapeutics
Source: Sci Rep. 2025 Feb 4;15:4198. doi: 10.1038/s41598-025-87316-w (PMC11794604; doi:10.1038/s41598-025-87316-w)
Supplement: Supplementary file 1 — Supplementary Information. [file 41598_2025_87316_MOESM1_ESM.docx]

**Development of an mPBPK Machine Learning Framework for Early Target Pharmacology Assessment of Biotherapeutics**

Krutika Patidar^1^, Nikhil Pillai^2^, Saroj Dhakal^2^, Lindsay B. Avery^3^, Panteleimon D. Mavroudis^2, *^

Affiliations:

^1^Department of Chemical and Biological Engineering, University at Buffalo, The State University of New York,
Buffalo, NY, USA

^2^Global DMPK Modeling & Simulation, Sanofi, Cambridge, MA, USA

^3^Global DMPK Innovation, Sanofi, Cambridge, MA, USA

^*^Corresponding author:

Panteleimon D. Mavroudis

350 Water St, Cambridge, MA-02141, USA

[panteleimon.mavroudis@sanofi.com](mailto:panteleimon.mavroudis@sanofi.com)

**Supplementary Information**

**Minimal PBPK model description**

The structure of the mPBPK model, a schematic diagram of which is shown in [1]. It comprises plasma, lymph, and two lumped tissue compartments, tight and leaky. Tight tissue represents muscle, fat, brain, and skin, whereas leaky tissue the rest of the body tissues. Arterial blood flow to tight/leaky tissue compartments is dependent on the vascular reflection coefficient for each tissue ($\sigma_{1}, \sigma_{2}$). The lymph flow out of each tissue compartment is collected in the lymph compartment, which is dependent on the lymphatic reflection coefficient ($\sigma_{L}$). The lymph flow rate to each tissue compartment ($L_{1}, L_{2}$) is calculated as a sum of individual tissue lymph flow rates reported previously [1]. Lymph flow from tight/leaky tissues is delivered back to plasma via the lymph compartment.

Each tissue is divided into vascular, endosomal, and interstitial spaces. The unbound drug and drug-target complex drains from the interstitial space into the lymph compartment and is delivered back to the plasma and the systemic circulation. Soluble targets are assumed to be synthesized both in plasma and in vascular space in the tissues, whereas membrane-bound targets are assumed to be synthesized in the tissue vascular space. The free mAb (A) binds to the soluble target (T) in plasma to form a soluble drug-target complex. The free mAb, target, and the A-T complex are taken up via pinocytosis into the nested endosomal sub-space in plasma, where A and A-T complex can bind to FcRn receptor at slightly acidic pH (pH=6) in the endosomes. The FcRn-bound mAb and FcRn-bound mAb-target complex are recycled back to plasma and salvaged from lysosomal degradation. The unbound mAb within the endosomes is degraded via a first-order rate constant by lysosomal degradation, whereas unbound T and A-T complex is catabolized in the endosomes. The unbound mAb and mAb-target complex circulate to the tissue compartments and enter the vascular sub-space in the tissues. The unbound drug can bind competitively with soluble and membrane-bound targets. The membrane-bound mAb-target complex is assumed to internalize at the same rate as the rate of target degradation as drug often times does not affect complex internalization [1]. The soluble mAb-target complex is taken up by tissue endosomes via pinocytosis. In the tissue endosomes, the unbound mAb and mAb-target complex interact with FcRn. The FcRn-bound mAb and FcRn-bound mAb-target complex are recycled back to both vascular space and interstitial space of the tissues [1].

**Supplementary Tables**

Table S1: List of mPBPK model simulations to obtain PK endpoints for virtual drug-target candidates (number of candidates, n = 10000).

| Simulation ID | Charge | Dose (mg/kg) | Regimen | Receptor Form | Criterion |
| --- | --- | --- | --- | --- | --- |
| 1 | 0 | 0.1 | Bolus (IV) | Soluble | $TO_{\max}$ |
| 2 | +5 | 0.1 | Bolus (IV) | Soluble | $TO_{\max}$ |
| 3 | -5 | 0.1 | Bolus (IV) | Soluble | $TO_{\max}$ |
| 4 | 0 | 0.1 | Bolus (IV) | Membrane | $TO_{\max}$ |
| 5 | +5 | 0.1 | Bolus (IV) | Membrane | $TO_{\max}$ |
| 6 | -5 | 0.1 | Bolus (IV) | Membrane | $TO_{\max}$ |
| 7 | 0 | 1 | Bolus (IV) | Soluble | $TO_{\max}$ |
| 8 | +5 | 1 | Bolus (IV) | Soluble | $TO_{\max}$ |
| 9 | -5 | 1 | Bolus (IV) | Soluble | $TO_{\max}$ |
| 10 | 0 | 1 | Bolus (IV) | Membrane | $TO_{\max}$ |
| 11 | +5 | 1 | Bolus (IV) | Membrane | $TO_{\max}$ |
| 12 | -5 | 1 | Bolus (IV) | Membrane | $TO_{\max}$ |
| 13 | 0 | 10 | Bolus (IV) | Soluble | $TO_{\max}$ |
| 14 | +5 | 10 | Bolus (IV) | Soluble | $TO_{\max}$ |
| 15 | -5 | 10 | Bolus (IV) | Soluble | $TO_{\max}$ |
| 16 | 0 | 10 | Bolus (IV) | Membrane | $TO_{\max}$ |
| 17 | +5 | 10 | Bolus (IV) | Membrane | $TO_{\max}$ |
| 18 | -5 | 10 | Bolus (IV) | Membrane | $TO_{\max}$ |
| 19 | 0 | 0.1 | Q2W (IV) | Soluble | $TO_{\mathrm{last}}$ |
| 20 | 0 | 1 | Q2W (IV) | Soluble | $TO_{\mathrm{last}}$ |
| 21 | 0 | 10 | Q2W (IV) | Soluble | $TO_{\mathrm{last}}$ |
| 22 | 0 | 1 | Q2W (IV) | Membrane | $TO_{\mathrm{last}}$ |
| 23 | +5 | 1 | Q2W (IV) | Soluble | $TO_{\mathrm{last}}$ |
| 24 | -5 | 1 | Q2W (IV) | Soluble | $TO_{\mathrm{last}}$ |
| 25 | 0 | 1 | Q1W (IV) | Soluble | $TO_{\mathrm{last}}$ |
| 26 | 0 | 1 | Q4W (IV) | Soluble | $TO_{\mathrm{last}}$ |

Charge: net surface charge on antibody candidate. Dose: administered antibody dose. Regimen: dosing scheme. Criterion: PK endpoint used for ML classification. IV: intravenous. Q1W: dose administered every 1 week. Q2W: dose administered every 2 weeks. Q4W: dose administered every 4 weeks. $TO_{\max}:$ target occupancy (TO) % calculated at maximum drug concentration. $TO_{\min}:$ target occupancy (TO) % calculated at minimum drug concentration.

Table S2: ML-derived rules for different scenarios using mPBPK model and Monolix/Simulix.

|  | **Candidate Properties** | | |  |  |  | $\mathbf{T}\mathbf{O}_{\mathbf{Cmin, plasma}}$ | | |
| --- | --- | --- | --- | --- | --- | --- | --- | --- | --- |
| ID | $T_{0}$  (nM) | $t_{1/2}$ (h) | $K_{D}$ (nM) | Dose (mg/kg) | Scheme | Charge | Monolix/Simulix | mPBPK model | ML prediction |
| 1 | 20 | 12 | 25 | 1 | Q2W | 0 | 65.4 % | 66.4 % | < 90 % |
| 2 | 5 | 15 | 12 | 1 | Q1W | 0 | 93.1 % | 91.4 % | > 90 % |
| 3 | 1 | 48 | 2 | 1 | Q4W | 0 | 93.2 % | 95 % | > 90 % |
| 4 | 0.05 | 1 | 5 | 0.1 | Q2W | 0 | 63 % | 61.6 % | < 90 % |
| 5 | 12 | 10 | 24 | 10 | Q2W | 0 | 96.7 % | 96.5 % | > 90 % |
| 6 | 15 | 6 | 15 | 1 | Q2W | 0 | 73.7 % | 72 % | 50-90 % |
| 7 | 10 | 12 | 2 | 1 | Q2W | 0 | 88.8 % | 94 % | > 90 % |

$T_{0}$ : Target baseline, $t_{1/2}$: Target half-life, $K_{D}$: Drug-target binding constant, Q1W: once every week, Q2W: once every two weeks, Q4W: once every 4 weeks, $TO_{Cmin, plasma}$: Target occupancy (%) calculated at Cmin in plasma.

Table S3: Experimental data for monoclonal antibodies.

| Drug | Target | Binding constant [nM] | Target Baseline [nM] | Target Half-Life [hr] | Clinical Dose / Regimen (Route) | Assumption*  (Dose / Regimen) | Source |
| --- | --- | --- | --- | --- | --- | --- | --- |
| Lebrikizumab | IL-13 | 0.0068 | 0.0013 | 0.25 | 1.8 mg/kg Q4W (IV) | 1 Q4W | [2, 3] |
| Anifrolumab | IFNAR | 0.067 | 0.0999 | 0.21 | 300 mg Q4W (IV) | 1 Q4W | [4] |
| Rituximab | CD20 | 8 | 0.320 | 426 | 1000 mg (IV) Q2W | 10 Q2W | [5, 6, 7] |
| Abciximab | GPIIb/IIIa | 0.0411 | 29.8 | 0.18 | 0.25 mg/kg (IV) bolus | 0.1 bolus | [8, 9, 10] |
| Basiliximab | CD25 | 1.2 | 4.8 | 6.39 | 20 mg (IV) bolus | 0.1 bolus | [11, 12] |
| Aducanumab | tau | 0.131 | 27 | 736.8 | 150 mg (IV) Q4W | 1 Q4W | [13] |
| Bevacizumab | VEGF-A | 0.058 | 11.3 | 345 | 5 mg/kg (IV) Q2W | 1 Q2W | [14, 15] |

*For simplicity, dose assumptions are made to match the dose and regimen used for trained ML model.

Table S4: Comparison of appropriate virtual data sample size based on test set accuracy and decision tree-based rules for each case.$T_{s0}$ is the target baseline (nM), $t_{1/2}$is the target half-life (hour), $K_{D}$ is the drug-target binding constant (nM).

|  | Sample size: 5000 | | | Sample size: 10000 | | | Sample size: 15000 | | |
| --- | --- | --- | --- | --- | --- | --- | --- | --- | --- |
|  | $T_{s0}$ | $t_{1/2}$ | $K_{D}$ | $T_{s0}$ | $t_{1/2}$ | $K_{D}$ | $T_{s0}$ | $t_{1/2}$ | $K_{D}$ |
| Rules | < 1.38 | < 89 | < 0.24 | < 1.33 | < 85 | < 0.15 | < 1.38 | < 114 | < 0.12 |
| Testing accuracy | 99.45 % (2356 samples) | | | 99.70% (4280 samples) | | | 99.54% (6306 samples) | | |

Table S5: Example of ML training dataset. Only a subset of candidates is shown.

| ID | Target baseline ($\mathbf{T}_{\mathbf{0}}$)  [nM] | Target half-life ($\mathbf{t}_{\mathbf{1/2}}$)  [hr] | Drug’s binding constant ($\mathbf{K}_{\mathbf{D}}$)  [nM] | Target Occupancy (TO)  [1: $\boldsymbol{>}$90%, 0: $\boldsymbol{\leq}$90%] |
| --- | --- | --- | --- | --- |
| 1 | 2.42 | 16.89 | 0.005 | 1 |
| 2 | 496.86 | 0.07 | 0.11 | 0 |
| 3 | 55.77 | 200.50 | 15.34 | 0 |
| 4 | 297.76 | 0.96 | 1.27 | 0 |
| 5 | 4.02 | 5.08 | 0.005 | 1 |

Table S6: Model performance evaluation across decision tree, random forest, and gradient boosting classifiers. Training metrics such as accuracy, precision, F1 score are mean % for 5-fold cross validation when training each algorithm. The standard deviation (SD) for each fold cross-validation is provided in braces. The testing metric evaluates model performance on the test dataset.

|  | | **Training Metric** | | | |
| --- | --- | --- | --- | --- | --- |
| **Algorithm** | | **Accuracy (SD)** | **Precision (SD)** | **F1-score (SD)** | **Time (s)** |
| Decision Trees | | 98.8 (0.002) | 98.3 (0.0017) | 98.8 (0.002) | 0.0330 |
| Random Forest | | 98.9 (0.002) | 98.7 (0.0019) | 98.8 (0.002) | 1.3069 |
| Gradient Boosting | | 99.2 (0.0018) | 98.9 (0.003) | 99.2 (0.002) | 2.4278 |
|  | **Testing Metric** | | | |  |
| **Algorithm** | | **Accuracy** | **Precision** | **F1-score** |  |
| Decision Trees | | 98.7 | 98.7 | 98.4 |  |
| Random Forest | | 97 | 97.1 | 95.1 |  |
| Gradient Boosting | | 99 | 99 | 98.9 |  |


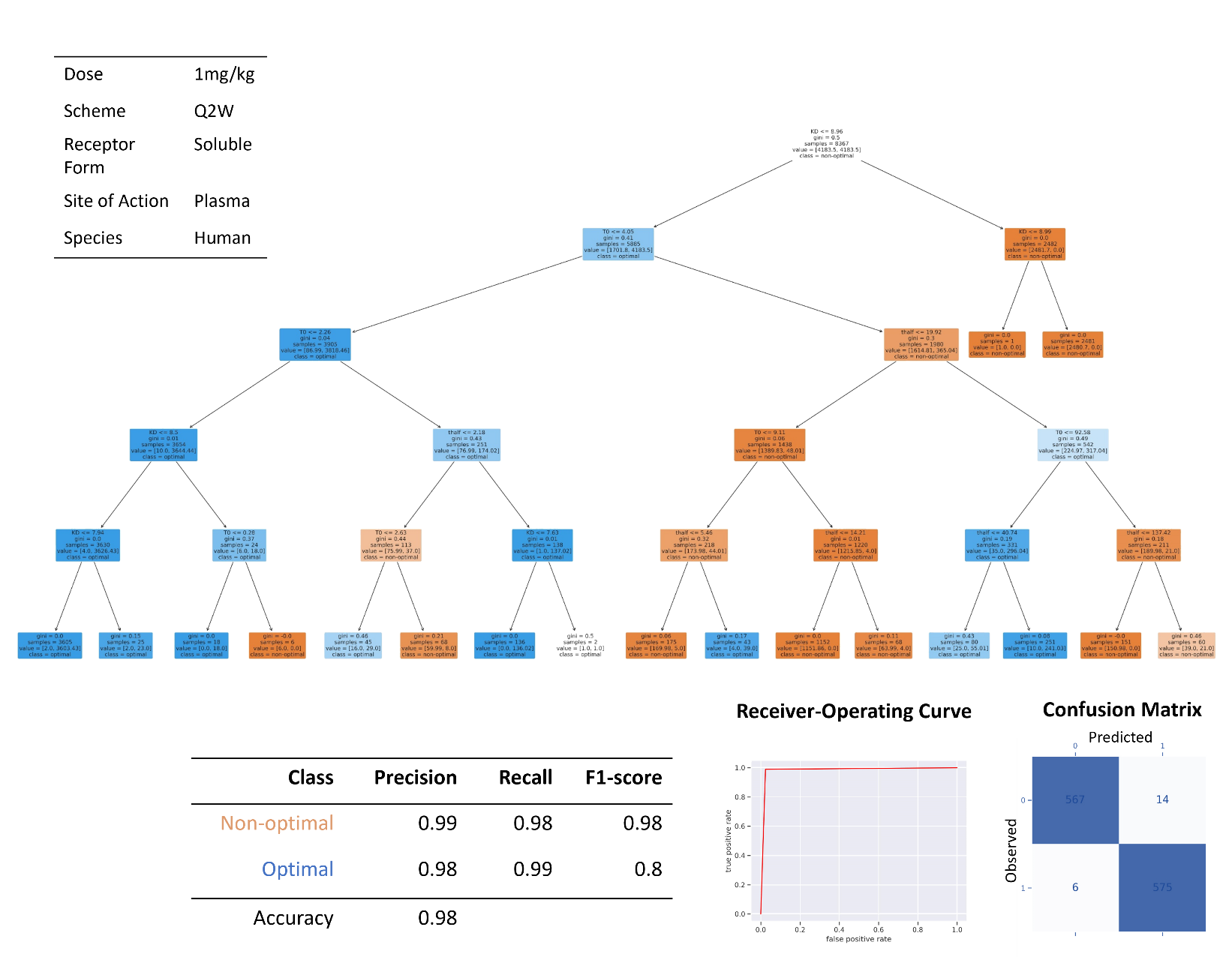
**Supplementary Figures/Data**

Figure S1: Summary of results for scenario 1. Antibody is administered at 1 mg/kg through IV route once every two weeks. Target Occupancy (TO %) is measured in plasma for drug and soluble target interaction. Decision tree classifier is trained for this scenario (top right). Classification report presents the precision, recall, F1-score, and accuracy of non-optimal and optimal class prediction. Receiver operating curve (ROC) and Confusion matrix (bottom right) depicts the model performance of the classifier.


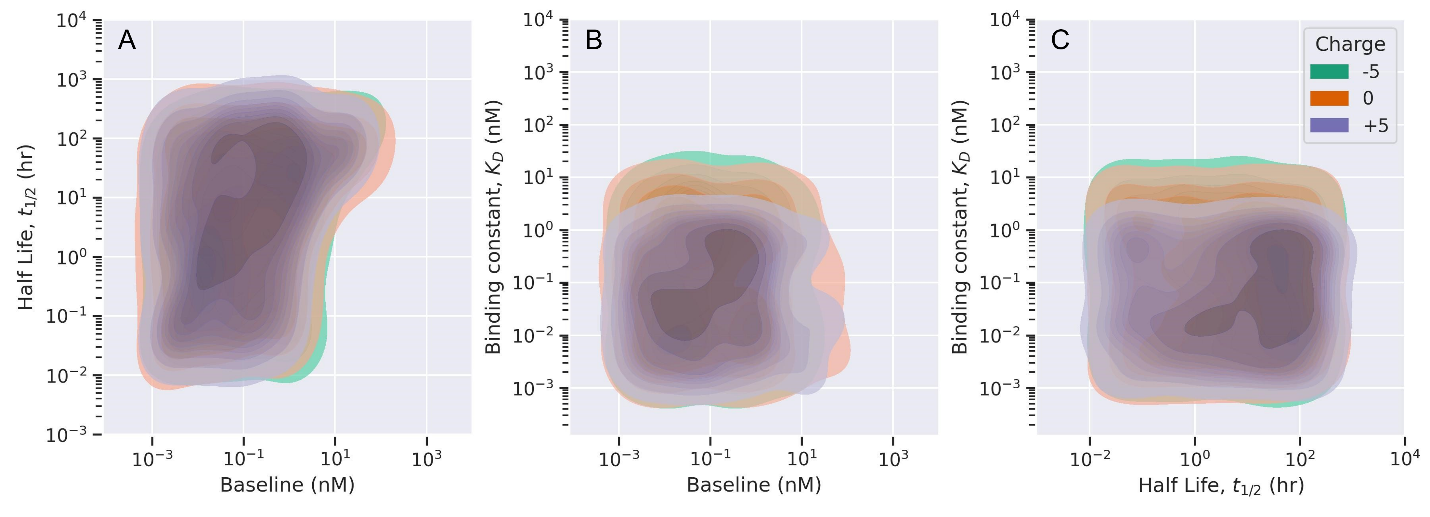


Figure S2: Pairwise density plot shows the effect of charge on optimal properties needed for greater than 90% target occupancy.


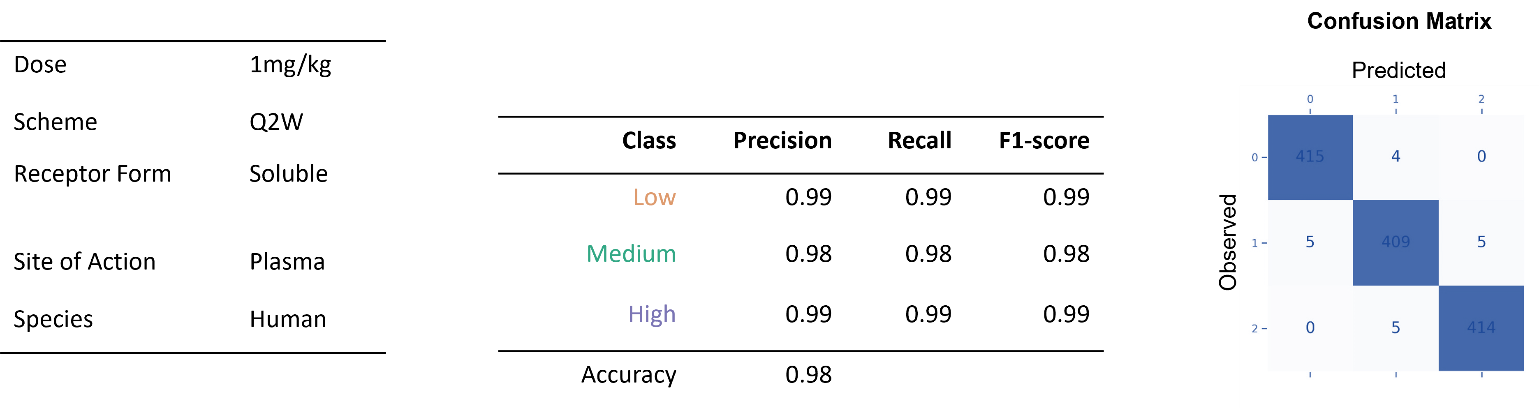
**Optimal rules derived from ML based on the scenario shown in Fig S2**

Figure S3: Summary of results for multi-label classification. Antibody is administered at 1 mg/kg through IV route once every two weeks. Target Occupancy (TO %) is measured in plasma for drug and soluble target interaction. Classification report (middle) presents the precision, recall, F1-score, and accuracy of low TO%, medium TO%, and high TO% class prediction. Confusion matrix (right) depicts the model performance of the classifier.

if (KD <= 8.965) and (T0 <= 4.047) and (T0 <= 2.261) and (KD <= 8.503) and (KD <= 7.943) then class: optimal (proba: 99.94%) | based on 3,605 samples

if (KD > 8.965) and (KD > 8.988) then class: non-optimal (proba: 100.0%) | based on 2,481 samples

if (KD <= 8.965) and (T0 > 4.047) and (thalf <= 19.916) and (T0 > 9.114) and (thalf <= 14.208) then class: non-optimal (proba: 100.0%) | based on 1,152 samples

if (KD <= 8.965) and (T0 > 4.047) and (thalf > 19.916) and (T0 <= 92.58) and (thalf > 40.74) then class: optimal (proba: 96.02%) | based on 251 samples

if (KD <= 8.965) and (T0 > 4.047) and (thalf <= 19.916) and (T0 <= 9.114) and (thalf <= 5.457) then class: non-optimal (proba: 97.14%) | based on 175 samples

if (KD <= 8.965) and (T0 > 4.047) and (thalf > 19.916) and (T0 > 92.58) and (thalf <= 137.423) then class: non-optimal (proba: 100.0%) | based on 151 samples

if (KD <= 8.965) and (T0 <= 4.047) and (T0 > 2.261) and (thalf > 2.182) and (KD <= 7.626) then class: optimal (proba: 100.0%) | based on 136 samples

if (KD <= 8.965) and (T0 > 4.047) and (thalf > 19.916) and (T0 <= 92.58) and (thalf <= 40.74) then class: optimal (proba: 68.76%) | based on 80 samples

if (KD <= 8.965) and (T0 > 4.047) and (thalf <= 19.916) and (T0 > 9.114) and (thalf > 14.208) then class: non-optimal (proba: 94.12%) | based on 68 samples

if (KD <= 8.965) and (T0 <= 4.047) and (T0 > 2.261) and (thalf <= 2.182) and (T0 > 2.628) then class: non-optimal (proba: 88.23%) | based on 68 samples

if (KD <= 8.965) and (T0 > 4.047) and (thalf > 19.916) and (T0 > 92.58) and (thalf > 137.423) then class: non-optimal (proba: 64.99%) | based on 60 samples

if (KD 8.965) and (T0 <= 4.047) and (T0 > 2.261) and (thalf <= 2.182) and (T0 <= 2.628) then class: optimal (proba: 64.45%) | based on 45 samples

if (KD <= 8.965) and (T0 > 4.047) and (thalf <= 19.916) and (T0 <= 9.114) and (thalf > 5.457) then class: optimal (proba: 90.7%) | based on 43 samples

if (KD <= 8.965) and (T0 <= 4.047) and (T0 <= 2.261) and (KD <= 8.503) and (KD > 7.943) then class: opti<= mal (proba: 92.0%) | based on 25 samples

if (KD <= 8.965) and (T0 <= 4.047) and (T0 <= 2.261) and (KD > 8.503) and (T0 <= 0.28) then class: optimal (proba: 100.0%) | based on 18 samples

if (KD <= 8.965) and (T0 <= 4.047) and (T0 <= 2.261) and (KD > 8.503) and (T0 > 0.28) then class: non-optimal (proba: 100.0%) | based on 6 samples

if (KD <= 8.965) and (T0 <= 4.047) and (T0 > 2.261) and (thalf > 2.182) and (KD > 7.626) then class: optimal (proba: 50.01%) | based on 2 samples

**Calculation of target occupancy (TO%)**

$\mathrm{TO}_{p} \left( \% \right)=\frac{C_{p,ATC}}{C_{p,ATC}+C_{p,T}}$ (S1)

where $\mathrm{TO}\left( \% \right)$ is the target occupancy % in plasma, $C_{\mathrm{ATC}}$ is the concentration of drug-target complex in plasma and $C_{p,T}$ is the concentration of target in plasma.

# References

| [1] | K. Patidar, N. Pillai, S. Dhakal , L. B. Avery and P. D. Mavroudis, "A minimal physiologically based pharmacokinetic model to study the combined effect of antibody size, charge, and binding affinity to FcRn/antigen on antibody pharmacokinetics.," *J Pharmacokinet Pharmacodyn.,* 2024. |
| --- | --- |
| [2] | Z. Tsilogianni, G. Hillas, P. Bakakos, L. Aggelakis, E. Konstantellou, A. L. Papaioannou, A. Papaporfyriou, S. Papiris, N. Koulouris, S. Loukides and K. Kostikas, "Sputum interleukin-13 as a biomarker for the evaluation of asthma control," *Clin Exp Allergy,* vol. 46, pp. 923-31, 2016. |
| [3] | A. J. Okragly, A. Ryuzoji, I. Wulur, M. Daniels, R. D. Van Horn, C. N. Patel and R. J. Benschop, "Binding, Neutralization and Internalization of the Interleukin-13 Antibody, Lebrikizumab," *Dermatology and Therapy,* vol. 13, pp. 1535-1547, 2023. |
| [4] | L. Peng, V. Oganesyan, H. Wu, W. F. Dall'Acqua and M. M. Damschroder, "Molecular basis for antagonistic activity of anifrolumab, an anti-interferon-α receptor 1 antibody," *MAbs,* vol. 7, pp. 428-39, 2015. |
| [5] | P. M. Glassman and J. P. Balthasar, "Physiologically-based modeling to predict the clinical behavior of monoclonal antibodies directed against lymphocyte antigens," *MAbs,* vol. 9, pp. 297-306, 2017. |
| [6] | M. D. Pescovitz, "Rituximab, an anti-cd20 monoclonal antibody: history and mechanism of action," *Am J Transplant,* vol. 6, pp. 859-66, 2006. |
| [7] | Biogen and Genentech USA, Inc., "Rituxan Prescribing Information," 1997. [Online]. Available: https://www.accessdata.fda.gov/drugsatfda_docs/label/2021/103705s5464lbl.pdf. [Accessed January 2024]. |
| [8] | D. E. Mager, M. A. Mascelli, N. S. Kleiman, D. J. Fitzgerald and D. R. Abernethy, "Simultaneous modeling of abciximab plasma concentrations and ex vivo pharmacodynamics in patients undergoing coronary angioplasty," *J Pharmacol Exp Ther,* vol. 307, pp. 969-76, 2003. |
| [9] | T. D. Liu, S. H. Ren, X. Ding, Z. L. Xie and Y. Kong, "A Short Half-Life alpha(IIb)beta(3) Antagonist ANTP266 Reduces Thrombus Formation," *Int J Mol Sci,* vol. 19, 2018. |
| [10] | J. P. Cunha, "ReoPro (Abciximab) drug," 2022. [Online]. Available: https://www.rxlist.com/reopro-drug.htm#description. [Accessed January 2024]. |
| [11] | O. Le Tilly, P. Gatault, C. Baron, T. Bejan-Angoulvant, M. Buchler, G. Paintaud and D. Ternant, "Is CD25 blockade optimal in kidney transplant patients treated with basiliximab? A target-mediated drug disposition model," *Br J Clin Pharmacol,* vol. 88, pp. 3500-3505, 2022. |
| [12] | Novartis Pharmaceutical Corp, "Simulect (basiliximab) Prescribing Information," 2003. [Online]. Available: https://www.accessdata.fda.gov/drugsatfda_docs/label/2003/basnov010203lb.htm. [Accessed January 2024]. |
| [13] | P. Bloomingdale, D. Bumbaca-Yadav, J. Sugam, S. Grauer, B. Smith, S. Antonenko, M. Judo, G. Azadi and K. L. Yee, "PBPK-PD modeling for the preclinical development and clinical translation of tau antibodies for Alzheimer's disease," *Front Pharmacol,* vol. 13, p. 867457, 2022. |
| [14] | S. Basu, Y. T. K. Lien, V. Vozmediano, J. F. Schlender, T. Eissing, S. Schmidt and C. Niederalt, "Physiologically Based Pharmacokinetic Modeling of Monoclonal Antibodies in Pediatric Populations Using PK-Sim," *Front Pharmacol,* vol. 11, p. 868, 2020. |
| [15] | Genentech, Inc., "Avastin (Bevacizumab) Prescribing Information," 2004. [Online]. Available: https://www.accessdata.fda.gov/drugsatfda_docs/label/2014/125085s301lbl.pdf. [Accessed January 2024]. |
